# Career Performance Trajectories in Track and Field Jumping Events from Youth to Senior Success: The Importance of Learning and Development

**DOI:** 10.1371/journal.pone.0170744

**Published:** 2017-01-27

**Authors:** Gennaro Boccia, Paolo Moisè, Alberto Franceschi, Francesco Trova, Davide Panero, Antonio La Torre, Alberto Rainoldi, Federico Schena, Marco Cardinale

**Affiliations:** 1 CeRiSM Research Center “Sport, Mountain, and Health”, Rovereto, (TN), Italy; 2 Motor Science Research Center, School of Exercise & Sport Sciences, SUISM, Department of Medical Sciences, University of Turin, Turin, Italy; 3 School of Exercise & Sport Sciences, SUISM, University of Turin, Turin, Italy; 4 School of Sport and Exercise Sciences, Department of Neurological and Movement Sciences, University of Verona, Verona, Italy; 5 Department of Biomedical Sciences for Health, Università degli Studi di Milano, Milan, Italy; 6 Aspire Academy, Doha, Qatar; 7 University College London, Department of Computer Science, London, United Kingdom; 8 University of St. Mark & St. John, Plymouth, United Kingdom; Nanyang Technological University, SINGAPORE

## Abstract

**Introduction:**

The idea that early sport success can be detrimental for long-term sport performance is still under debate. Therefore, the aims of this study were to examine the career trajectories of Italian high and long jumpers to provide a better understanding of performance development in jumping events.

**Methods:**

The official long-jump and high-jump rankings of the Italian Track and Field Federation were collected from the age of 12 to career termination, for both genders from the year 1994 to 2014. Top-level athletes were identified as those with a percentile of their personal best performance between 97 and 100.

**Results:**

The age of entering competitions of top-level athletes was not different than the rest of the athletic population, whereas top-level athletes performed their personal best later than the rest of the athletes. Top-level athletes showed an overall higher rate of improvement in performance from the age of 13 to the age of 18 years when compared to all other individuals. Only 10–25% of the top-level adult athletes were top-level at the age of 16. Around 60% of the top-level young at the age of 16 did not maintain the same level of performance in adulthood. Female high-jump represented an exception from this trend since in this group most top-level young become top-level adult athletes.

**Conclusions:**

These findings suggest that performance before the age of 16 is not a good predictor of adult performance in long and high jump. The annual rate of improvements from 13 to 18 years should be included as a predictor of success rather than performance per se. Coaches should be careful about predicting future success based on performances obtained during youth in jumping events.

## Introduction

Sports academies and talent identification programs aimed at developing sporting talent have increased in number and resources over recent years [1,2] mainly due to the recognized need to develop talent pathways to guarantee success on the international stage [3]. Various approaches have been used to detect, confirm and develop talent mostly based on multidisciplinary assessments but many are still strongly biased towards competition results, in particular in CGS (sports measured in centimeters, grams, or seconds) sports. A comprehensive definition of “sports talent” is lacking, however it is accepted in the literature and in sporting environments [4] that a talent in sports is an individual whose athletic performances are superior to his peer group and is capable of reaching or has achieved consisting performances at top level. Few authors have in fact argued the case for late developers [5] due to the fact that bias towards results at the early stage of the athletic career might exclude late maturing children and/or children capable of long lasting high performance due to the multidimensional nature of talent development [6] and the need to take into consideration psychological and learning aspects [7,8]. In sports where the performance outcome is clearly defined like CGS talent identification and confirmation is usually based on tracking the development of specific physical traits and their manifestation in the performance arena. Running, jumping and throwing are skills determined to a certain extent by genetic factors [9–12] and developed through life with training as well as being affected by growth and maturation [13,14].

Both male and female athletes benefit from training and competitive environments and are able to improve performance during childhood in running and jumping events with men outperforming women to a larger extent near or after puberty [15,16]. Recent work from Tonnesen et al. [15] in particular showed that male athletes start to outperform females in jumping and running after the age of 12 with jumping events results showing an accelerated improvement up to 14 years of age (in males) to then show a very slow improvement by age 18 in males and a plateau by age 16 in females. Overall, the study from Tonnesen indicated that from the age of 11 to the age of 18 Norwegian boys improve high jump and long jump performances by 41% and 48% and girls by 24% and 26%. Despite the highly technical demands of both jumping events, the performance improvements are clearly parallel to the development of strength and power typical of growth and maturation stages from childhood to adulthood. Previous studies have in fact extensively reported that vertical jumping and horizontal jumping ability is similar in boys and girls before puberty [17,18]. However, despite the fact that it is well established that jumping abilities develop with age, plateau with adulthood [19] and decline with ageing [20] and retain marked gender differences, there is a lack of studies on the development of high and long jump performances during the competitive lifespan of athletes with particular reference to elite vs. non elite performers. Such information could be used to establish progression ranges to help coaches and administrators in tracking athletes development with realistic expectations, as already evidenced in Swimming [21]. While the recent work from Tonnesen and coworkers has provided more clarity over the expected progression and differences in high and long jump performances from the age of 11 to 18, less is known about the implications of such progressions for performance in adulthood, as well as differences between the development of jumping skills in elite and non-elite performers. While growth and maturation are important aspects affecting performance in young age, other aspects such as training and competition quality, coaching and family support and socio-economic environment have a strong influence [22]. Talent expression is in fact acknowledged to be determined by complex interactions between various aspects, however this would require a comprehensive integrative approach to study a large number of athletes over a long period of time. To our knowledge, no studies to date have investigated expected annual performance developments in jumping disciplines among competitive athletes and presented the differences between top-performers and the rest. Furthermore, information regarding the realistic rate of development for a dedicated athlete from the start to the end of the performance career is currently lacking in the research literature and as a consequence there is a lack of reference to appropriate development pathways for young athletes. The Italian Athletics Federation (FIDAL) has over several years recorded official competitions results for all Italian athletes entering competitions from the age of 12. Access to such databases provide the opportunity to interrogate the development of athletic performance from childhood to retirement and therefore allow the possibility to study and quantify progressions of performance in high and long jump with high accuracy.

Considering the lack of studies in this field and the need to provide more evidence to talent confirmation and development for coaches and administrators we structured and interrogated the results database to answer the following specific questions. We focused the investigation on high and long jump disciplines of the track and field: 1) Does the age of entering competition influence the personal best performance? 2) Do the top-level athletes reach their personal best performance at the same age of the others? 3) Does the annual rate of change of performance in young ages differ from top level to other individuals? 4) How were the top-level adult performed when they were young? 5) How top level youth performed when they became adult? 6) Is it possible to predict the personal best performance by an adult by knowing the performance by young athlete?

We hypothesized that: 1) the age of entering competition would not be a predictor of top-level performance; 2) top-level athletes would reach their personal best later than others; 3) top-level athletes would show greater annual rate of change of performance; 4) performance at young age would not predict performance at senior level.

## Materials and Methods

### Data collection

This retrospective study was conducted in accordance with the declaration of Helsinki. Since our data are based on publicly available resources, no informed consent was obtained. This study was approved by the local ethics committee of the University of Verona. The Italian Track and Field Federation (FIDAL) annually publishes all-time best results categorized by genders, age, and discipline. For this study we retrieved all data from High Jump and Long Jump competitions. The high jump is a track and field event in which competitors must jump unaided over a horizontal bar placed at measured heights without dislodging it. The long jump is a track and field event in which athletes perform a run in an attempt to leap as far as possible from a take-off point marked on a track before a sandpit used for landing. The raw data set are presented in a table which reports performance, name, wind velocity, birth year, club, competition date and venue where the result was set. The database is restricted to the individual seasonal best for each discipline and data were available from 1994 either in paper or electronic form. In the present study, the 200 annual best athletes from 1994 to 2014 were included in the analysis. From 1994 to 2004 data were available only in paper documents which were converted in digital worksheets by two of the authors. From the 2005 to 2014 data were available on the online database of the FIDAL website (www.fidal.it). The rankings were collected from the age of 12 to 35, or to 31/12/2014 if the individual is still active in two jumping events: long and high jump. For each individual, the year of entering competition and career termination was defined as the first and last appearance in the recorded ranks, respectively. Only results obtained with legal wind speed (≤2 m/s) were included for analysis.

### Data processing

Longitudinal data of each individual were extrapolated by custom-written software in MATLAB R2014a (Mathworks, Natick, Massachusetts). Separate analyses were performed by discipline and gender. Records were included only if the individuals were present in the rankings for almost three years, also none consecutively. The individuals of each discipline were ranked on the basis of the percentile of the absolute value of their personal best performance. Therefore, all available personal best performances of each discipline were included in an “all-time” ranking including the records from the years 1994 to 2014. Individuals were then sub-grouped in 25 subgroups based on the percentile of their personal best performance. Each subgroup consisted in four percentiles of the sample, from the worst performer, with a percentile between 1 and 4, to the best group (top-level), with a percentile between 97 and 100. In order to analyze the placement of the individuals during adolescence, the “all-time” ranking based on the percentiles, and the successive sub-grouping, were also performed for each year of youth. Herein we arbitrarily use the term youth to identify age from 12 to 17, and adult for age equal or higher than 18.

The annual rate of change of performance, the age of entering competition, and the age of personal best performance were identified for each individual and averaged for each subgroup. The youth annual rates of change of performance were compared between top-level athletes and the rest of the sample using a two-way ANOVA (2 group x 6 age), with successive post-hoc *t* tests with Bonferroni correction. The age of entering competition and the age of best performance between top-level athletes and the other subgroups were compared using one-way ANOVA with Dunnet’s post hoc tests.

We identified how many top-level adult athletes were considered top-level when performing in youth categories and how many top-level young individuals became adult top-level adult athletes. The Pearson’s *r* correlation coefficient between paired performances throughout the ages was determined as normative stability data. This statistical procedure was previously used for longitudinal analysis of young swimmers performances [23]. The stability was considered to be high if *r*≥0.60, moderate if 0.30≤*r*<0.60, and low if *r*<0.30 as suggested by Malina [24]. To assess the usefulness of introducing the annual rate of change of performance to predict the adult personal best performance we compared the goodness of prediction (*R*^*2*^) of two regression models. The first model was a regression analysis including as an independent factor only the annual best performances. Whereas the second model consisted in a multiple regression analysis including as independent factors both the annual best performances and the annual rate of change of performance. Both analytical approaches were applied for each age from 13 to 18 years to predict personal best performance. The significance level was set at *p* <0.05. The Statistical Package for Social Sciences (SPSS 20.0 for Windows) was used for all statistical analyses.

## Results

### Identification of top-level athletes

**Fig 1:**
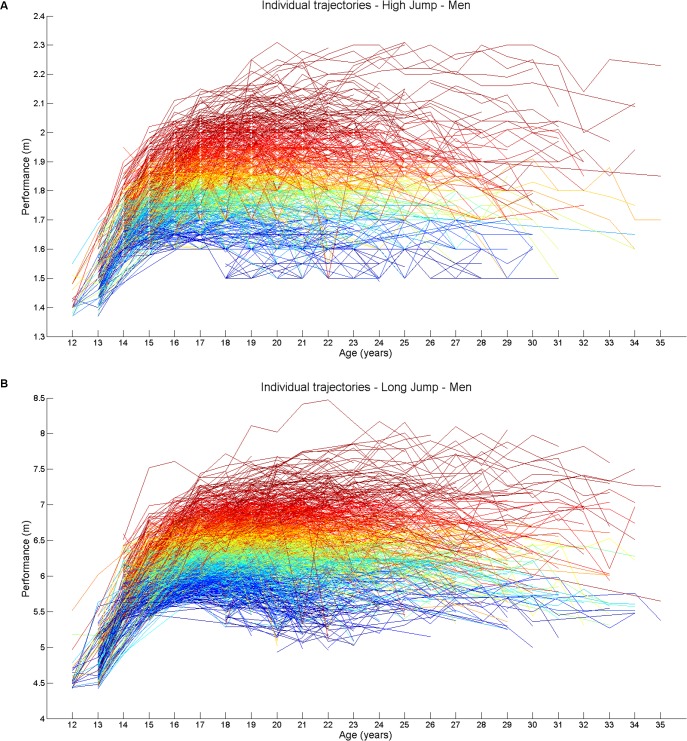
**Individual performance trajectories in males (A) high jump and (B) long jump athletes.** The individual trajectories are reported for each athlete with color varying according to personal best performance, from blue (lowest level athletes) to red (highest level athlete).

Overall, the database contained (after error and duplication removal) records from 1266 male high jumpers, 1442 male long jumpers, 898 female high jumpers, and 1258 female long jumpers. The individuals belonging to the top-level groups were: 60 male high jumpers with best performance higher than 2.10 m, 67 male long jumpers with best performance higher than 7.35 m, 42 female high jumpers with best performance higher than 1.78 m, 63 female long jumpers with best performance higher than 5.97 m. Representative examples of individual performance trajectories are reported in Fig 1A for male high jump and in Fig 1B for male long jump.

### Age of entering competitions

The age of entering competition was not different across subgroups of performances in any disciplines and genders. The age of entering competition: was 15.5±2.4 years for top men high jumpers, with no difference with respect to the other subgroups (F = 0.87, p = 0.55); was 15.4±1.4 years top men long jumpers, with no difference with respect to the other subgroups (F = 0.48, p = 0.89); 14.4±1.2 years for top women high jumpers, with no difference with respect to the other subgroups (F = 1.1, p = 0.09); 14.6±1.3 years for top women long jumpers, with no difference with respect to the other subgroups (F = 0.92, p = 0.50).

### Age of personal best

**Fig 2:**
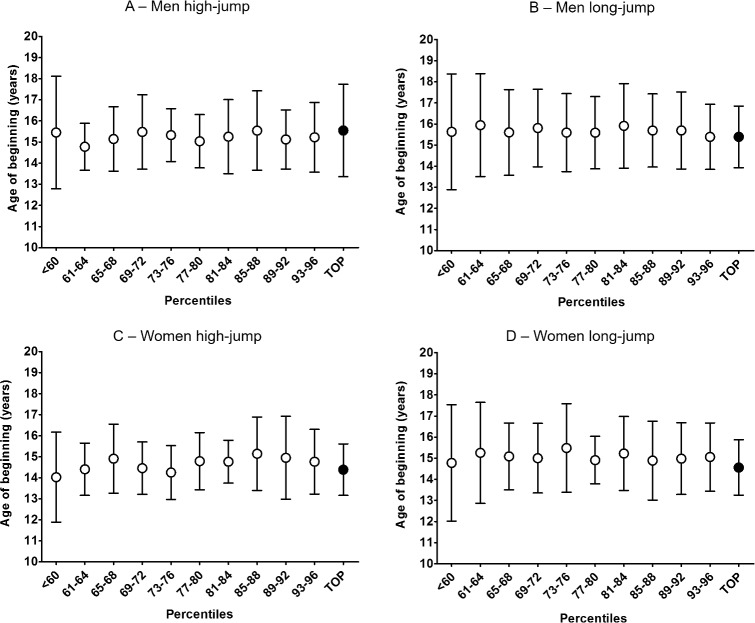
Age reaching personal best performance. The age of reaching personal best performance (mean±SD) is reported for each subgroup of athlete. The sample was sub-grouped on the base of the percentiles of the personal best performance (reported in the x axis). Overall, top-level athletes reached their personal best performance later than the rest of the sample in all disciplines and genders: (A) men high jump; (B) men long jump; (C) women high jump; (D) women long jump. Post hoc analysis is showed as: *** p<0.0001.

Fig 2 shows the ages of achieving personal best performance for each disciplines and the post-hoc analysis: overall, the age of personal best performance of top-level athletes was higher than that of other subgroups. The age of personal best performance: in top-level male high jumpers was 21.6±2.6 years and was statistically significant higher (F = 17.8, p<0.0001) than in other subgroups; in top-level male long jumpers the age of personal best was 23.4±3.2 years, and was statistically significant higher (F = 17.3, p<0.0001) than in all other subgroups (Fig 2B); in top-level female high jumpers the age of personal best was 21.1±2.9 years, and was statistically significant higher (F = 19.5, p<0.0001) than in all other subgroups (Fig 2C); in top-level female long jumpers was 21.5±3.1 years, and was statistically higher (F = 18.9, p<0.0001) than in all subgroups other than the 93–96 percentiles group (Fig 2D).

### Annual rate of change in performance

**Fig 3:**
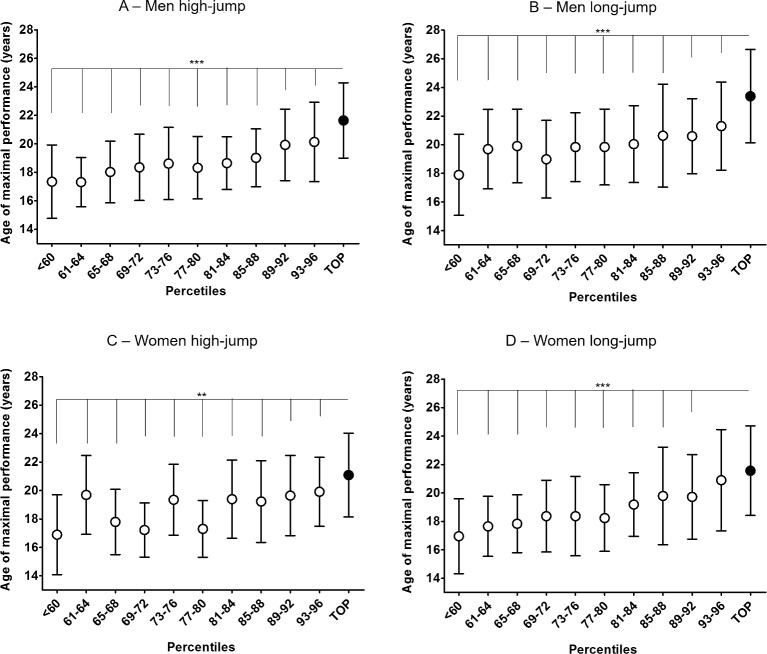
Annual rate of change in performance. For each age from 14 to 18 years, the annual rates of change in performance (mean±SD) are reported for top-level athletes and the rest of the sample. Overall, top-level athletes showed greater annual rate of change in performance than the rest of the sample in all disciplines and genders: (A) men high jump; (B) men long jump; (C) women high jump; (D) women long jump. Post hoc analysis are reported as *p<0.05; **p<0.01; **p<0.001.

The Fig 3 shows the comparisons between the rates of change in performance between top-level athletes and the rest of the sample. The group × age interaction was found to be statistically significant only for male long-jump (F = 2.6, p = 0.02) while being non-significant for all other disciplines (all *p* values > 0.18). The top-level group showed greater rate of change in performance than the rest of the sample in all disciplines and genders (main effect for group: all *p* values < 0.005). In general, the rate of change in performance decreases with increasing age in the whole sample and all disciplines and genders (main effect for age: all *p* values < 0.0001). The results of the post hoc analysis are reported in Fig 3.

### Youth performance of adult top-level athletes

In the Table 1 are reported, for each age of analysis, the percentage of top-level adult athletes that was considered top-level when they were young. The following ratios identify the top-level adult athletes that started their competitions later than 18 years old: 6/60 (10%) male high jumpers, 5/67 (7%) male long jumpers, 1/42 (2%) female high jumpers, and 2/63 (3%) female long jumpers.

**Table 1:** Percentages of top-level adult athletes that were considered top-level when they were young.

Groups	Age (years)					
	12	13	14	15	16	17
**Men–High-jump**	0	2	3	16	25	40
**Men–Long-jump**	0	0	4	14	10	29
**Women–High-jump**	2	2	21	47	59	59
**Women–Long-jump**	0	5	14	22	25	36
90% confidence limits	from 12% to 25%					

For each age from 12 to 17, each cell represents the percentage of the top-level adult athletes who were top-level young performer.

### Adult performances of top-level young individuals

In the Table 2 are reported, for each age of analysis, the percentage of top-level young that became top-level athletes in the adulthood.

**Table 2:** Percentages of top-level young that became top-level in the adulthood.

Groups	Age (years)					
	12	13	14	15	16	17
**Men–High-jump**	0	0	5	25	41	59
**Men–Long-jump**	0	0	21	30	25	57
**Women–High-jump**	10	13	50	63	89	79
**Women–Long-jump**	0	38	40	36	40	63
90% confidence limits	from 23% to 42%					

For each age from 12 to 17, each cell represents the percentage of top level young that performed at top level when they became adult.

In the S1 Table are reported the correlation coefficients for pair-wise ages and for personal best performance. At younger ages there was lower performance correlation coefficients when considering its progression to adulthood. Stabilization is considered when correlation coefficients between the actual performances and the best performances was higher than 0.60. High stability of performance in our sample is achieved at 16 years in men high-jump (r = 0.66) and long-jump (r = 0.60), at 15 years in women high-jump (r = 0.66) an at 16 years in women long-jump (r = 0.60).

**Fig 4:**
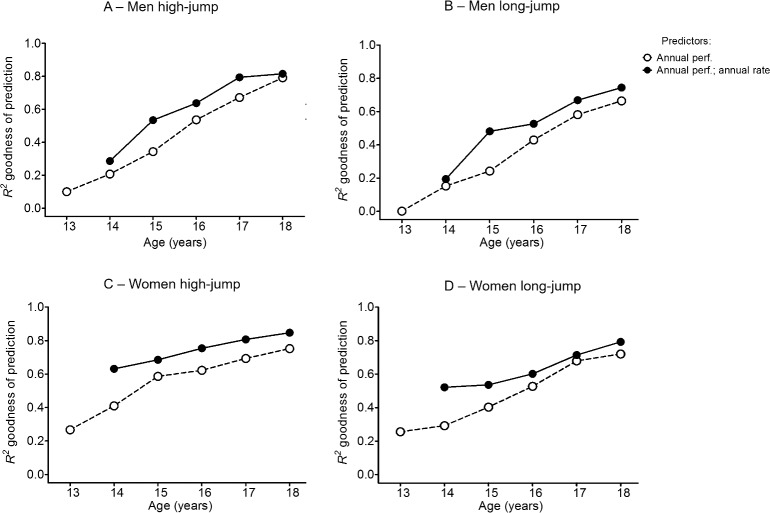
Goodness personal best prediction. In the Fig 4 are reported the R^2^ coefficients, as indices of prediction goodness, resulted from the multiple regression analysis conducted from 13 to 18 years old. When both annual best performance and annual rate of change were included in the regression model as independent factors (filled circles) the goodness of prediction was consistently greater than using only annual best performance as independent factor (empty circles). The annual rate of change from 12 to 13 years are not reported because the samples were too small to perform the analysis. The data are reported for men high-jump (A), men long-jump (C), women high-jump (C) and women long-jump (D) groups.

The results of multiple regression analyses are reported in the S2 Table. Based on partial correlation coefficients *R*, both the annual best performance and the annual rate of change of performance positively correlated with the personal best performance for all ages from 13 to 18 years. The *R*^*2*^ coefficients increased throughout the youth (from 13 to 18 years old). Importantly, based on *R*^*2*^ results, the inclusion of the annual rate of change as independent factor, improved the goodness of the personal best prediction. This trend was consistent across all ages of analysis and disciplines, see Fig 4.

## Discussion

To the authors’ knowledge, this is the first study to present absolute and relative annual performance developments in jumping events for competitive athletes from early adolescence to career termination in a large population. Due to the observational and cross-sectional study design, interpretation of the present results is limited to some extent, however it provides the opportunity to quantify and describe the typical developments of male and female athletes in two athletic disciplines. Similar work recently published by Tønnessen et al. [25] focused only on the best 100 Norwegian athletes and looked at the age range between 11 to 18 years. Similar to our data, relative performance development was highest at age 13–14 and declined gradually when reaching adulthood in both male and female athletes in high and long jump. In women’s high jump, the rate of development was very limited from age 16 with a small difference between the top group and the rest of the population (Cohen’s *d* = 0.10–0.32), see Fig 3C. In the long jump a similar trend was observed with less than 1% performance improvements from age 16 to over 19 (Cohen’s *d* = 0.34–0.50), see Fig 3D. A main difference was evident from male athletes, which still seem to improve up to older ages. In particular, the top group still progresses quite remarkably in both high and long jump (Fig 3A and 3B). While such differences between males and females results can be explained by the differences in growth and maturation between genders, one cannot dismiss the technical development aspects and/or the quality of coaching input. It is very well established that jumping performance is affected by age [26,27], gender [16–18,28] and activity levels [29]. In particular, fat free mass differences post peak height velocity between male and females [19,30] as well as the differences in the utilization of stored elastic energy in the leg extensors muscles highlighted in earlier work [28] and the contribution of testosterone and muscle fibers composition in leg extensors [31,32] might explain the differences in jumping abilities. The results of our study suggest that high jump and long jump performances progress to a similar degree to jumping abilities measured with typical field tests like counter movement jumps and/or standing long jumps. In fact, the percentage improvements in high jump and long jump performances are similar to the improvements in the jumping abilities assessed with field tests in various populations from the ages of 13 to 16 [18,28,33,34] clearly suggesting the strong influence of growth and maturation in this age range. However, technical development and coaching high and long jump should take place in a structured manner during the growth and maturation development. In fact, as the top-level group shows larger improvements than 97% of the population, it is likely that this group of athletes, apart from possible better neuromuscular qualities, might be more capable of learning and improving their technical abilities leading to higher and longer jumps. Since we had no access to any physical performance data, it is impossible to assess if the top-level population had superior neuromuscular qualities than 97% of the athletes analyzed in this study and associating performance only to muscle strength would be an inaccurate reflection of the complexity of high and long jump performance. Motor learning and skill acquisition as well as coaching should be brought into the equation. Gender differences in motor learning cannot be excluded as mean nerve conduction velocity in the Central Nervous System has been shown to be faster in young adult males [35] and brain maturation and development has been suggested to be affected by growth and gender [36]. However, in general, gender differences in motor learning have been mostly ascribed to environmental factors [37] even if some experimental evidence seems to suggest differences in performances depending on the tasks studied [38–40]. As there is no longitudinal study available to make inferences about gender differences in learning complex motor skills, the data in our study can constitute the basis for a re-evaluation of some of the assumptions of development of technical coaching for male and female athletes. In light of our data, we think that a parallel assessment of technical development and motor learning, paired with the continuous assessment of physical abilities is the most appropriate approach to assess talent development in jumping events.

The remarkable reduction in the rate of improvement in high and long jump performances in adulthood observed in our cohort (Fig 3) also suggest that the improvement possibilities are very limited once growth and maturation is completed and therefore coaching interventions need to be “smarter”. While we don’t know the training history and activities of the cohorts analyzed, the fact that the athletes were still actively competing indicates that at least they were performing technical training to improve high and long jump and while the male top 4% of the cohort still showed improvements between age 18–19, this was not evident in the female cohort.

The starting age of entering competition showed no difference between the top-level group and the rest of the population in both men and women in both athletic events. This suggests that in these two disciplines in our population the top-level group started training and competition activities at the same time as the athletes which did not reach elite performances in adult age. Therefore, while in many coaching communities there is the belief that early specialization might be an advantage in jumping disciplines, our data don’t seem to provide support to advantage of an early start. This is further supported by a recent meta-analysis which suggested no difference in starting age of training and competition in highly skilled athletes as compared to less skilled athletes [41]. Future studies should explore this aspect in other sports and disciplines, but our data support the view that in these two highly technical events early specialization would not be beneficial to obtain better results in senior competitions.

The age at which the top-level group reached the personal best was higher in both events and for both gender, clearly indicating the continuation of practice and possibly the ability to stay injury-free is a major determinant for elite performances immediately after junior competitions (Fig 2). This is supported by our analyses of the presence of the top-level senior performers in top groups as youths. In both men and women, it was clear that only after puberty there is a significant presence of athletes in the top-level group senior performers but only for a limited percentage. For example, only 10–25% of top-level adult were in the top-level group at the age of 16. The lack of evidence for early specialization is also supported by a small percentage of athlete reaching the top-level level of performance despite entering competition at 18 years of age of after. Moreover, around 60–75% of the top-level young at the age of 16 did not maintain the same level of performance in adulthood. Female high-jumpers represented an exception from this trend since in this group most top-level young become top-level adult athletes indicating that in this discipline performances at junior level might be better predictors of senior performances.

When considering the correlation coefficients between performances throughout the youth ages year by year (S1 Table) it is possible to assess the performance stability across the youth career [23][24]. Proceeding diagonally through the table, the coefficients tended to be higher with increasing age when considered, suggesting an increase in performance stability with increasing age. This means that with increasing ages the performance is progressively more predictable. This result was similar to that found in young freestyle swimmers [22]. When considering the correlation coefficients between the performances throughout the youth ages and personal best performance (horizontal line of S1 Table) it is possible to see an increase between the ages of 12 to 18. In males, the coefficients become high (i.e. *r* > 0.60) from the age of 16 in both disciplines. In females, the correlations reached the *r*>0.60 at the age of 15 and 16 for high- and long-jump, respectively. These findings confirm that the predictability of adult performances from youth performance is problematic before the age of 15–16 years and talent identification based only actual performance data cannot thus be performed before this age.

Previous work on throwing events has already questioned the ability to predict senior performances from performances in junior World Championships [42] and our work supports the view that a systematic approach to tracking athletes development in the context of realistic expected improvements with growth should prevail over sport dogma. Using multiple regression analysis (S2 Table), attempts to predict adult performances improve accuracy when considering both the annual rate of change and the absolute performance (Fig 4). This is a novel and important finding since it suggests the inclusion of annual rate of change, calculated across the youth, as a good predictor for future performances. However, still a large variance in personal bests remains unexplained at the age of 18 (adjusted *R*^*2*^ 0.79–0.88). What was clear, was that the rate of improvement of the top-level group was larger at young age than the rest of the population (Fig 3) and therefore a multiannual assessment of youth athletes should consider more the rate of improvement before puberty rather than absolute performances which should be looked with more attention after puberty. This is possibly due to the fact that top-level performers are better at learning the motor skills required in these two athletic events than the rest of the population even if their absolute performances at young ages do not put them in the top group. However not for all this is true, as we have seen a small percentage of top performers able to enter competitions at age 18 or older and still reach the top-level group as adults. This reinforces the need to understand that predicting adult performances is very difficult in particular when growth, development and most of all learning aspects are not taken into account [43]. Furthermore, our data suggest that early specialization in these two athletic disciplines may not be conducive to reaching elite performance as it is possible that a more diverse sporting exposure may be more productive before the age of 15 [44]. Finally, as other authors have suggested the need to assess and consider psychological characteristics of developing excellence like commitment, self-belief and competitiveness [45], one should not exclude the positive reward of the rate of improvement at the young age as another aspect facilitating retention in the sport and success later in the sporting career.

When interpreting the current data, it should be recognized that the threshold used to define top-level athletes, as those who performed in the best 4% of the distribution, may affect the results. However, we modeled the data after assessing that the significance of the study did not substantially change by identifying top-level between 5% and 1% of the distribution. Moreover there are some limitations associated with using ratios and percentage change values [46]. For this reason, we used mixed model analyses on absolute rather than ratio values and clustered the population by percentiles to identify the differences between the top 4% and the rest. Furthermore, percentage change and ratio data are presented supplemental to absolute values and effect size statistics was also introduced to quantify the magnitude of progressions and differences.

Despite the limitations in the data due to the absence of maturation and growth status of the individuals in the database we believe this information is very important for coaches, clubs and governing bodies in order to track appropriately the performance development of young athletes and benchmark their progressions. Furthermore, our data can provide a term of reference when setting performance expectations on young athletes suggesting that before the age of 16 talent identification cannot be performed. Finally, we hope these data will be used to establish ways to assess and ascertain the effectiveness of coaching interventions as well as providing an avenue to conduct more research into the technical coaching aspects of these track and field events in order to develop appropriate coaching methodologies for each age group.

### Conclusions

These findings suggest that performance before the age of 16 is not a good predictor of adult performance in long and high jump. The rate of improvements from 13 to 18 years provide important indication of performance progression and should be considered more favorably when assessing career potential in young athletes. Moreover, most of the top-level adult athlete were not top-level at youth level and most of the top-level youth did not maintain the same level of performance in adulthood. Longitudinal performance assessment combined with growth and maturation assessment should be implemented when assessing talent progression.

## Supporting Information

S1 Table Pearson correlation coefficients throughout the youth performance careers. In the S1 Table are reported the correlation coefficients for pair-wise ages and for personal best performance.
(DOCX)

S2 Table Regression analysis to predict adult best performance. In the S1 Table are reported the estimates of the independent factors annual best performance and annual rate of change for the prediction of the adult personal best performances.
(DOCX)
