# Correction: Career Performance Trajectories in Track and Field Jumping Events from Youth to Senior Success: The Importance of Learning and Development

**DOI:** 10.1371/journal.pone.0178662

**Published:** 2017-05-23

**Authors:** Gennaro Boccia, Paolo Moisè, Alberto Franceseschi, Francesco Trova, Davide Panero, Antonio La Torre, Alberto Rainoldi, Federico Schena, Marco Cardinale

Fig 2 appears incorrectly. Please see the correct Fig 2 and its figure legend here.

**Fig 2:**
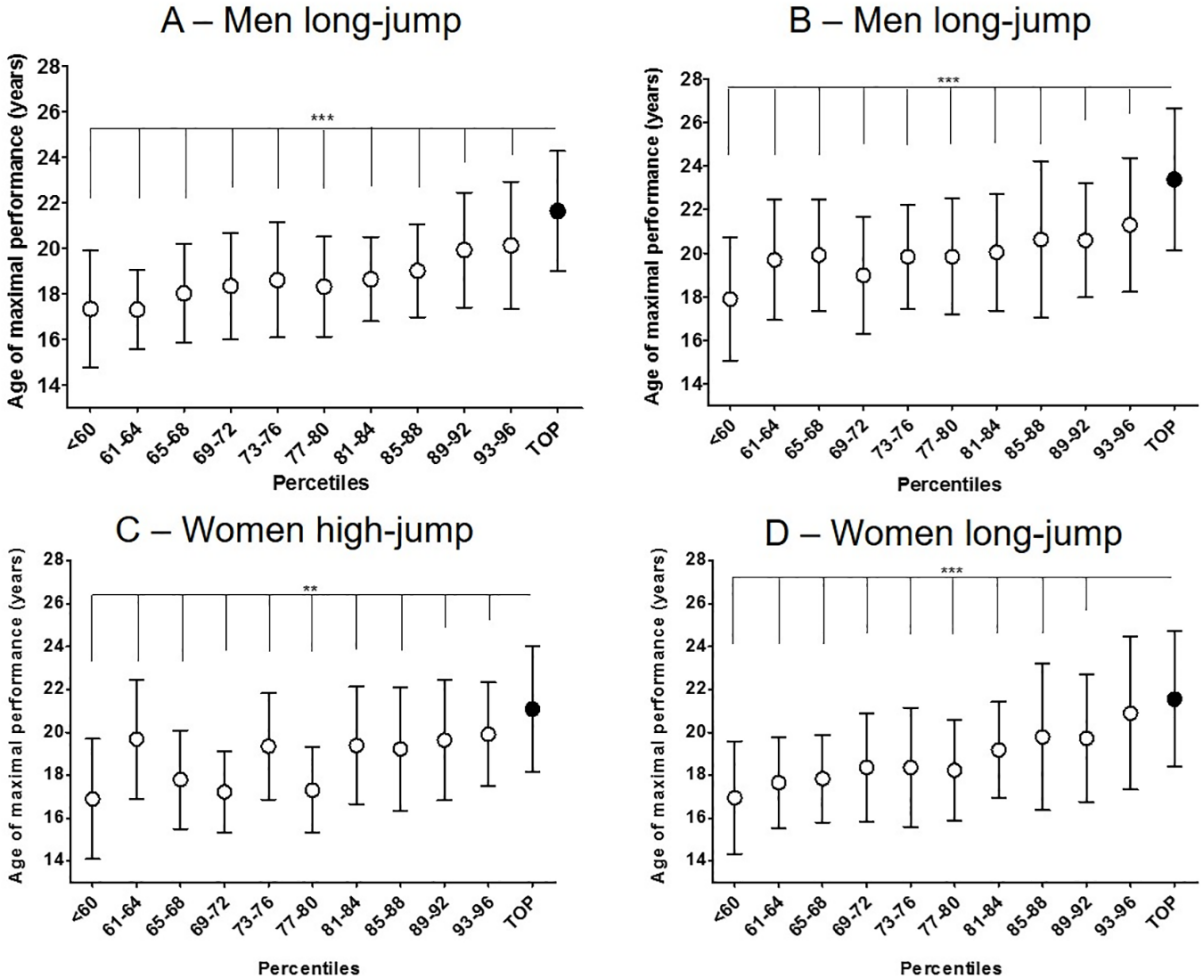
Age reaching personal best performance. The age of reaching personal best performance (mean±SD) is reported for each subgroup of athlete. The sample was sub-grouped on the base of the percentiles of the personal best performance (reported in the x axis). Overall, top-level athletes reached their personal best performance later than the rest of the sample in all disciplines and genders: (A) men high jump; (B) men long jump; (C) women high jump; (D) women long jump. Post hoc analysis is showed as: *** p<0.0001.

Fig 3 appears incorrectly. Please see the correct Fig 3 and its figure legend here.

**Fig 3:**
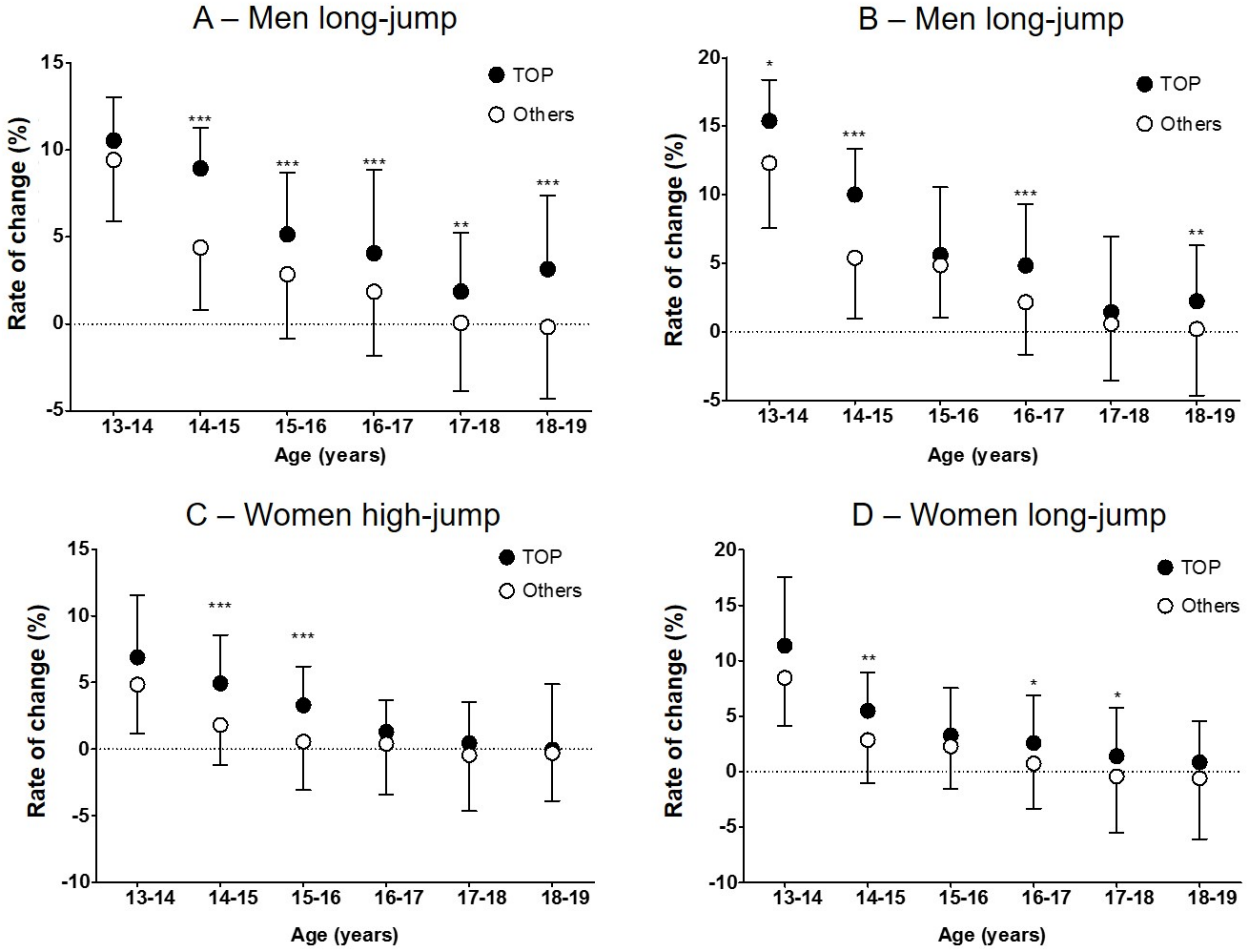
Annual rate of change in performance. For each age from 14 to 18 years, the annual rates of change in performance (mean±SD) are reported for top-level athletes and the rest of the sample. Overall, top-level athletes showed greater annual rate of change in performance than the rest of the sample in all disciplines and genders: (A) men high jump; (B) men long jump; (C) women high jump; (D) women long jump. Post hoc analysis are reported as *p<0.05; **p<0.01; **p<0.001.
