# Supplementary material for: Career Performance Trajectories in Track and Field Jumping Events from Youth to Senior Success: The Importance of Learning and Development
Source: PLoS One. 2017 Jan 27;12(1):e0170744. doi: 10.1371/journal.pone.0170744 (PMC5271320; doi:10.1371/journal.pone.0170744)
Supplement: S1 Table — In the S1 Table are reported the correlation coefficients for pair-wise ages and for personal best performance. (DOCX) [file pone.0170744.s001.docx]

**S1 Table. Pearson correlation coefficients throughout the youth performance careers.**

In the S1 Table are reported the correlation coefficients for pair-wise ages and for personal best performance. At younger ages, there was lower performance correlation coefficients when considering its progression to adulthood. Stabilization is considered when correlation coefficients between the actual performances and the best performances is higher than 0.60. High stability of performance, see the horizontal line of correlation coefficients with the best performance, is achieved at 16 years in men high-jump (r=0.66) and long-jump (r=0.60), at 15 years in women high-jump (r=0.66) an at 16 years in women long-jump (r=0.60).

| **Men high-jump** |  | **Age (years)** | | | | | | |  |
| --- | --- | --- | --- | --- | --- | --- | --- | --- | --- |
|  | **Age (years)** | **12** | **13** | **14** | **15** | **16** | **17** | **18** | **Best** |
|  | **12** | 1 |  |  |  |  |  |  |  |
|  | **13** | 0.56^*^ | 1 |  |  |  |  |  |  |
|  | **14** | 0.44 | 0.66^**^ | 1 |  |  |  |  |  |
|  | **15** | 0.38 | 0.49^**^ | 0.68^**^ | 1 |  |  |  |  |
|  | **16** | 0.39 | 0.43^**^ | 0.58^**^ | 0.75^**^ | 1 |  |  |  |
|  | **17** | 0.40 | 0.38^**^ | 0.47^**^ | 0.62^**^ | 0.77^**^ | 1 |  |  |
|  | **18** | 0.65^*^ | 0.27^**^ | 0.34^**^ | 0.55^**^ | 0.69^**^ | 0.82^**^ | 1 |  |
|  | **Best** | -0.31 | 0.18 | 0.28^*^ | 0.47^**^ | 0.66^**^ | 0.75^**^ | 0.89^**^ | 1 |
|  |  |  |  |  |  |  |  |  |  |
| **Men long-jump** |  | **Age (years)** | | | | | | |  |
|  | **Age (years)** | **12** | **13** | **14** | **15** | **16** | **17** | **18** | **Best** |
|  | **12** | 1 |  |  |  |  |  |  |  |
|  | **13** | 0.46^*^ | 1 |  |  |  |  |  |  |
|  | **14** | 0.22 | 0.36^**^ | 1 |  |  |  |  |  |
|  | **15** | 0.45^*^ | 0.25^*^ | 0.54^**^ | 1 |  |  |  |  |
|  | **16** | 0.51^*^ | 0.24^*^ | 0.35^**^ | 0.68^**^ | 1 |  |  |  |
|  | **17** | 0.18 | 0.19 | 0.34^**^ | 0.59^**^ | 0.70^**^ | 1 |  |  |
|  | **18** | -0.02 | 0.00 | 0.41^**^ | 0.56^**^ | 0.66^**^ | 0.74^**^ | 1 |  |
|  | **Best** | 0.25 | -0.04 | 0.32^**^ | 0.44^**^ | 0.60^**^ | 0.65^**^ | 0.82^**^ | 1 |
|  |  |  |  |  |  |  |  |  |  |
| **Women high-jump** |  | **Age (years)** | | | | | | |  |
|  | **Age (years)** | **12** | **13** | **14** | **15** | **16** | **17** | **18** | **Best** |
|  | **12** | 1 |  |  |  |  |  |  |  |
|  | **13** | 0.50^**^ | 1 |  |  |  |  |  |  |
|  | **14** | 0.50^**^ | 0.57^**^ | 1 |  |  |  | ***­*** |  |
|  | **15** | 0.48^*^ | 0.53^**^ | 0.73^**^ | 1 |  |  |  |  |
|  | **16** | 0.54^*^ | 0.52^**^ | 0.59^**^ | 0.75^**^ | 1 |  |  |  |
|  | **17** | 0.43^*^ | 0.43^**^ | 0.52^**^ | 0.69^**^ | 0.79^**^ | 1 |  |  |
|  | **18** | 0.56^*^ | 0.45^**^ | 0.49^**^ | 0.64^**^ | 0.73^**^ | 0.77^**^ | 1 |  |
|  | **Best** | 0.54^*^ | 0.48^**^ | 0.51^**^ | 0.66^**^ | 0.70^**^ | 0.75^**^ | 0.91^**^ | 1 |
|  |  |  |  |  |  |  |  |  |  |
| **Women long-jump** |  | **Age (years)** | | | | | | |  |
|  | **Age (years)** | **12** | **13** | **14** | **15** | **16** | **17** | **18** | **Best** |
|  | **12** | 1 |  |  |  |  |  |  |  |
|  | **13** | 0.37^*^ | 1 |  |  |  |  |  |  |
|  | **14** | -0.05 | 0.47^**^ | 1 |  |  |  |  |  |
|  | **15** | 0.11 | 0.42^**^ | 0.65^**^ | 1 |  |  |  |  |
|  | **16** | -0.13 | 0.42^**^ | 0.53^**^ | 0.72^**^ | 1 |  |  |  |
|  | **17** | -0.15 | 0.45^**^ | 0.43^**^ | 0.60^**^ | 0.73^**^ | 1 |  |  |
|  | **18** | -0.28 | 0.48^**^ | 0.39^**^ | 0.55^**^ | 0.63^**^ | 0.74^**^ | 1 |  |
|  | **Best** | -0.19 | 0.52^**^ | 0.41^**^ | 0.51^**^ | 0.60^**^ | 0.70^**^ | 0.87^**^ | 1 |

*The p values of correlations are reported as* ^*^ *p<0.01,* ^**^ *p<0.0001.*
