# Supplementary material for: Career Performance Trajectories in Track and Field Jumping Events from Youth to Senior Success: The Importance of Learning and Development
Source: PLoS One. 2017 Jan 27;12(1):e0170744. doi: 10.1371/journal.pone.0170744 (PMC5271320; doi:10.1371/journal.pone.0170744)
Supplement: S2 Table — In the S1 Table are reported the estimates of the independent factors annual best performance and annual rate of change for the prediction of the adult personal best performances. (DOCX) [file pone.0170744.s002.docx]

| Table A – Multiple regression analysis to predict adult best performance | | | | | | | | | | | | | | | | |
| --- | --- | --- | --- | --- | --- | --- | --- | --- | --- | --- | --- | --- | --- | --- | --- | --- |
|  |  | Men – High-jump | | |  | Men – Long-jump | | |  | Women – High-jump | | |  | Women – Long-jump | | |
|  | Independent variables | *β* | Partial *R* | *R^2^* |  | *β* | Partial *R* | *R^2^* |  | *β* | Partial *R* | *R^2^* |  | *β* | Partial *R* | *R^2^* |
| Age 14 y |  |  |  | 0.286 |  |  |  | 0.194 |  |  |  | 0.631 |  |  |  | 0.522 |
|  | Perf. at 13 | 0.491 | 0.454^*^ |  |  | 0.205 | 0.349^*^ |  |  | 0.862 | 0.776^*^ |  |  | 0.837 | 0.714^*^ |  |
|  | Change 13−14 | 0.552 | 0.497^*^ |  |  | 0.206 | 0.440^*^ |  |  | 0.695 | 0.266^*^ |  |  | 0.613 | 0.598^*^ |  |
| Age 15 y |  |  |  | 0.534 |  |  |  | 0.526 |  |  |  | 0.685 |  |  |  | 0.536 |
|  | Perf. at 14 | 0.508 | 0.595^*^ |  |  | 0.515 | 0.591^*^ |  |  | 0.683 | 0.772^*^ |  |  | 0.628 | 0.672^*^ |  |
|  | Change 14−15 | 0.574 | 0.642^*^ |  |  | 0.625 | 0.665^*^ |  |  | 0.526 | 0.683^*^ |  |  | 0.501 | 0.587^*^ |  |
| Age 16 y |  |  |  | 0.637 |  |  |  | 0.481 |  |  |  | 0.754 |  |  |  | 0.602 |
|  | Perf. at 15 | 0.728 | 0.760^*^ |  |  | 0.739 | 0.675^*^ |  |  | 0.826 | 0.881^*^ |  |  | 0.737 | 0.751^*^ |  |
|  | Change 15−16 | 0.560 | 0.668^*^ |  |  | 0.548 | 0.562^*^ |  |  | 0.473 | 0.367^*^ |  |  | 0.457 | 0.577^*^ |  |
| Age 17 y |  |  |  | 0.794 |  |  |  | 0.668 |  |  |  | 0.807 |  |  |  | 0.714 |
|  | Perf. at 16 | 0.849 | 0.877^*^ |  |  | 0.745 | 0.786^*^ |  |  | 0.893 | 0.866^*^ |  |  | 0.830 | 0.834^*^ |  |
|  | Change 16−17 | 0.522 | 0.746^*^ |  |  | 0.497 | 0.647^*^ |  |  | 0.377 | 0.590^*^ |  |  | 0.445 | 0.629^*^ |  |
| Age 18 y |  |  |  | 0.815 |  |  |  | 0.744 |  |  |  | 0.846 |  |  |  | 0.793 |
|  | Perf. at 17 | 0.882 | 0.896^*^ |  |  | 0.841 | 0.852^*^ |  |  | 0.943 | 0.918^*^ |  |  | 0.866 | 0.884^*^ |  |
|  | Change 17−18 | 0.385 | 0.662^*^ |  |  | 0.411 | 0.623^*^ |  |  | 0.407 | 0.707^*^ |  |  | 0.341 | 0.597^*^ |  |
| *The p values of the partial R are reported as* ^*^ *p<0.001.* | | | | | | | | | | | | | | | | |

The S2 Table A shows the estimates of the independent factors a) annual best performance and b) annual rate of change for the prediction of the adult personal best performances, calculated for the ages from 14 to 18 years old. Based on partial correlation coefficients, the annual best performance and the annual rate of change of performance positively correlated with the personal best performance for all ages from 13 to 18 years. The *R^2^* coefficients increased throughout the youth (from 13 to 18 years old).

| Table B – Regression analysis to predict adult best performance | | | | | | | | | | | | | | | | |
| --- | --- | --- | --- | --- | --- | --- | --- | --- | --- | --- | --- | --- | --- | --- | --- | --- |
|  |  | Men – High-jump | | |  | Men – Long-jump | | |  | Women – High-jump | | |  | Women – Long-jump | | |
|  | Independent variables | *β* | Partial *R* | *R^2^* |  | *β* | Partial *R* | *R^2^* |  | *β* | Partial *R* | *R^2^* |  | *β* | Partial *R* | *R^2^* |
| Age 14 y |  |  |  |  |  |  |  |  |  |  |  |  |  |  |  |  |
|  | Perf. at 14 | 0.455 | 0.455^*^ | 0.207 |  | 0.389 | 0.389^*^ | 0.152 |  | 0.640 | 0.640^*^ | 0.410 |  | 0.540 | 0.540^*^ | 0.290 |
| Age 15 y |  |  |  |  |  |  |  |  |  |  |  |  |  |  |  |  |
|  | Perf. at 15 | 0.586 | 0.586^*^ | 0.343 |  | 0.492 | 0.492^*^ | 0.240 |  | 0.766 | 0.766^*^ | 0.586 |  | 0.635 | 0.635^*^ | 0.402 |
| Age 16 y |  |  |  |  |  |  |  |  |  |  |  |  |  |  |  |  |
|  | Perf. at 16 | 0.732 | 0.732^*^ | 0.536 |  | 0.655 | 0.655^*^ | 0.428 |  | 0.789 | 0.789^*^ | 0.622 |  | 0.726 | 0.726^*^ | 0.527 |
| Age 17 y |  |  |  |  |  |  |  |  |  |  |  |  |  |  |  |  |
|  | Perf. at 17 | 0.819 | 0.819^*^ | 0.671 |  | 0.762 | 0.762^*^ | 0.580 |  | 0.833 | 0.833^*^ | 0.693 |  | 0.824 | 0.824^*^ | 0.679 |
| Age 18 y |  |  |  |  |  |  |  |  |  |  |  |  |  |  |  |  |
|  | Perf. at 18 | 0.890 | 0.890^*^ | 0.791 |  | 0.815 | 0.815^*^ | 0.664 |  | 0.867 | 0.867^*^ | 0.752 |  | 0.848 | 0.848^*^ | 0.720 |
| *The p values of the partial R are reported as* ^*^ *p<0.001.* | | | | | | | | | | | | | | | | |

The S2 Table B shows the estimates of the independent factor annual best performance for the prediction of the adult personal best performances, calculated for the ages from 14 to 18 years old. Based on partial correlation coefficients, the annual best performance performance positively correlated with the personal best performance for all ages from 13 to 18 years. The *R^2^* coefficients increased throughout the youth (from 13 to 18 years old).
